# Observational learning computations in neurons of the human anterior cingulate cortex

**DOI:** 10.1038/ncomms12722

**Published:** 2016-09-06

**Authors:** Michael R. Hill, Erie D. Boorman, Itzhak Fried

**Affiliations:** 1 Division of Biology and Biological Engineering, California Institute of Technology, Pasadena, California 91125, USA; 2 Computation and Neural Systems, California Institute of Technology, Pasadena, California 91125, USA; 3 Division of Neurosurgery and Neuropsychiatric Institute, University of California (UCLA), Los Angeles, California 90095, USA; 4 Center for Neuroprosthetics, Ecole Polytechnique Fédérale de Lausanne (EPFL), Campus Biotech H4, Chemin des Mines 9, 1202 Genève, Switzerland; 5 Department of Humanities and Social Sciences, California Institute of Technology, Pasadena, California 91125, USA; 6 Centre for Functional Magnetic Resonance Imaging of the Brain, University of Oxford, John Radcliffe Hospital, Oxford OX3 9DU, UK; 7 Functional Neurosurgery Unit, Tel-Aviv Medical Centre and Sackler Faculty of Medicine, Tel-Aviv University, Tel-Aviv 69978, Israel

## Abstract

When learning from direct experience, neurons in the primate brain have been shown to encode a teaching signal used by algorithms in artificial intelligence: the reward prediction error (PE)—the difference between how rewarding an event is, and how rewarding it was expected to be. However, in humans and other species learning often takes place by observing other individuals. Here, we show that, when humans observe other players in a card game, neurons in their rostral anterior cingulate cortex (rACC) encode both the expected value of an observed choice, and the PE after the outcome was revealed. Notably, during the same task neurons recorded in the amygdala (AMY) and the rostromedial prefrontal cortex (rmPFC) do not exhibit this type of encoding. Our results suggest that humans learn by observing others, at least in part through the encoding of observational PEs in single neurons in the rACC.

## Abstract (web summary)

Humans can learn alone or by watching others, strategies which may depend on similar or different neural networks. This study shows that people watching other players in a card game used computations in neurons of their rostral anterior cingulate cortex to learn through observation.

## Graphical abstract

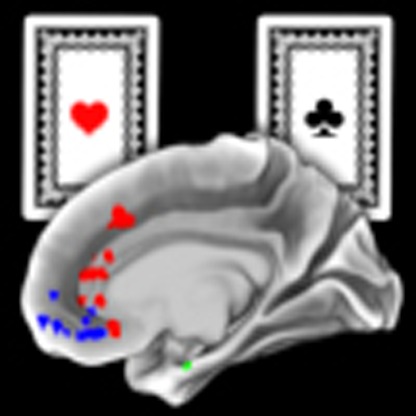

Reinforcement learning theory posits that learning can occur by means of calculating prediction errors (PEs)^1^. The seminal discovery that the phasic firing of dopamine neurons in the primate midbrain resembles the encoding of such PEs when animals learn from direct experience, fundamentally advanced our understanding of the neural mechanisms driving non-social reinforcement learning in primates^2^,^3^,^4^,^5^. More recent single-neuron studies in monkeys have also reported non-observational PEs in various regions outside of the dopaminergic midbrain, including the anterior cingulate cortex (ACC)^6^,^7^,^8^. The highly social structure of human societies, however, means that many of our decisions are influenced by choices and outcomes, which we observe in others. For example, we may choose to buy food from one street vendor over another because we have been observing a line of people making that same choice and enjoying good food as a result thereof. We may even survive a perilous situation only because we have observed others trying different escape routes with varying degrees of success before we choose the safest possible option for our own escape. Such observational learning is prevalent in many species, including octopi, rodents and primates, and is thought to form the basis of cultural diversification in animals and humans^9^,^10^,^11^; the computation underlying these fundamental behaviours, however, has so far not been described at the level of single neurons.

Guided by cross-species evidence of AMY, rostromedial prefrontal cortex (rmPFC) and ACC involvement in both social and reward-based processes^12^, we collected a unique dataset of single-neuron recordings in these three structures in humans to investigate whether they encode key computations predicted by formal learning theory but for learning through the observation of others. This approach also enabled us to compare the relative contributions of single neurons within these areas to learning in the same paradigm for the first time.

Subjects performed a novel card game task consisting of five rounds per game. In each round the subjects first played out one trial themselves (self-experienced) and then observed two other players play out the next two trials (observed; Fig. 1a). In each trial a player had to pick a card from either the left card deck of one suit (for example, clubs) or the right card deck of another suit (for example, spades Fig. 1b). The subjects were informed that one of the decks provided a 30%, and the other a 70% chance of drawing a winning card, and that, within the winning and losing cards, respectively, the amounts ($10 or $100) were distributed randomly and equally. The subjects were also advised that they could therefore learn which was the better deck by keeping track of their own, self-experienced win/lose outcomes as well as those of the other two observed players, who also picked their cards from the same decks. In each experimental session, after the card game task (12 games), the subjects also played a one-armed random slot machine task with a 50% chance of winning or losing (Fig. 1c).

The findings presented here show that neurons in the AMY and the rmPFC encode the outcome (winning or losing) for self-experienced trials and to a limited extent for observed trials, or alternatively for slot machine trials. Neurons in the rostral anterior cingulate cortex (rACC), however, encode all three trial types. In the card game neurons in all three brain areas also encode the amount won or lost, but only in the rACC are these parameters encoded differently for self-experience and observed trials. Finally, we show that a population of rACC neurons encode observational PEs. Taken together these findings emphasize the rACC's role in observational learning and provide the first single-cell evidence of the nature of the computation at work during these processes in humans.

## Results

### Behaviour

Subjects' choices in the card game were faster when choosing the objectively better deck as opposed to the worse deck (*P*<10^−5^, two-tailed *t*-test, *n*=1,268 and 592 trials, Supplementary Fig. 1a). They were faster following immediately previously observed wins compared with immediately previously observed losses (*P*=0.003<0.05, *t*-test, *n*=987 and 873, Supplementary Fig. 1b) and following increasing numbers of coherent previous outcomes (for example, the left card losing and the right card winning both coherently predict the right card deck to be the better one; *P*=0.016, Spearman test, *n*=1,860, Supplementary Fig. 1c). Previous outcomes in the chosen deck did not influence reaction times significantly (Supplementary Fig. 1d–f). Choice time was significantly higher during the first round of a game, when no prior knowledge was available (*P*<10^−5^, ANOVA, *n*=1,860) but not significantly different between subsequent rounds 2–5 (*P*>0.05, ANOVA, *n*=1,488, Supplementary Fig. 1g) and no effect on choice times was found for the side, from which a card was drawn (*P*>0.05, *t*-test, *n*=966 and 894, Supplementary Fig. 1h).

**Figure 1:**
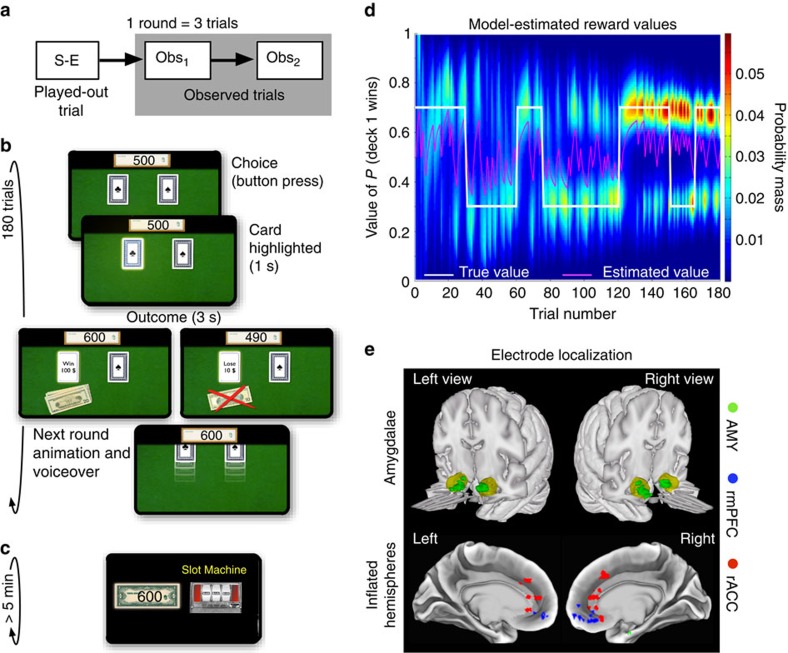
Behavioural task, model performance and electrode localization. (**a**) In the card game task subjects played 12 games consisting of 5 rounds each. Every round consisted of one self-experienced (S-E) trial, played out by the subject, and two observed trials (Obs_1_ and Obs_2_). (**b**) Structure of a trial in the card game task (self-experienced trials and observed trials had the same structure). (**c**) Screenshot of the slot machine task, which subjects played *ad libitum* for at least 5 min. (**d**) Estimated card deck reward values generated by the Bayesian learning model used to model subjects' expected value predictions and behavior. The heatmap depicts the probability mass, or relative likelihood, of each value of the distribution over reward values for deck 1 (arbitrarily defined) on each choice trial for an example subject. The true reward generating probability is shown in white. The mean of the distribution on each trial is shown in magenta, which forms our estimate of each participant's expected value. The true probabilities reversed with a probability of 50% at the beginning of each game (15 trials). (**e**) Estimated localization of the recording sites across all 10 subjects, projected onto the Caret Conte69 human surface-based atlas (MNI space, Supplementary Table 1).

For a more in depth analysis we also constructed a normative hierarchical Bayesian reversal-learning model (Fig. 1d and ‘Methods' section). The model-derived trial-by-trial difference in the expected values between card decks and the choice entropy (a measure of choice difficulty that captures the model-estimated uncertainty about a choice) both reliably predicted the likelihood that subjects would pick a card from a particular deck (logistic regression analysis predicting choices of the left deck over the right deck: expected value difference: *P*<10^−5^; choice entropy: *P*<0.005, one-tailed *t*-test, *n*=10 subjects). Furthermore, choice entropy also predicted how long subjects would take to make the respective choice (multiple linear regression predicting choice time: expected value difference: *P*>0.05; choice entropy: *P*<10^−5^, one-tailed *t*-test, *n*=10), indicating subjects' decisions were slower when the model predicted they were more difficult. These analyses confirm that the model quantitatively captured trial-by-trial variation in subject behaviour in this task (Supplementary Fig. 2a).

To test more directly whether subjects' choices were explained by past win/loss outcomes and prediction errors, we performed further regression analyses. These analyses showed that subjects did in fact learn from both the previous win/loss outcomes of their own self-experienced choices and those of the other players whose choices they observed (logistic regression analysis predicting subject choices on current trial *t* from the previous two win/loss outcomes of each player, spanning previous trials *t*-1 to *t*-6; one-tailed *t*-test, averaging over previous two choices for self-experienced trials: *P*=0.0006 and separately, for observed trials: *P*=0.0001, *n*=10; Supplementary Fig. 2b; see ‘Methods' section). The results from this analysis imply that subjects' choices were a function of prediction errors computed from both self-experienced and observed past outcomes. To more directly test this relationship, we used the full prediction error term [win/loss—choice expected value (computed from the reversal-learning model)] from the most recent past trial for both self-experienced and observed outcomes in the same regression model to predict subject choices in the current trial *t.* This analysis furnished strong evidence that subjects' choices in the current trial could indeed be predicted by the most recent self-experienced and observed prediction errors (self-experienced: *P*<10^−7^; observed: *P*<10^−5^, *n*=10; Supplementary Fig. 2c; see ‘Methods' section), thereby motivating our attempts to identify neuronal correlates of self-experienced and observational prediction errors in the human brain.

### Neuronal response properties

While subjects performed the experimental paradigm we recorded neuronal spiking activity using microwires implanted in their AMY, rmPFC and rACC^13^ (Fig. 1e). From 842 recorded units, we isolated 358 single neurons (42.5%, Supplementary Fig. 3) and all subsequent analysis was conducted using these single-units only (125 neurons in the AMY with a mean firing rate of *f*=2.51 +/− 0.22 Hz; 95 in the rmPFC, *f*=1.72 +/− 0.18 Hz; and 138 in the rACC, *f*=2.28 +/− 0.19 Hz; *f* was not found to be significantly different across areas in an ANOVA, *P*>0.05, *n*=358). During task performance the mean firing rate in all three brain areas was elevated, albeit non-significantly (*f*(AMY)=2.88 +/− 0.35 Hz, *f*(rmPFC)=2.2 +/− 0.35 Hz, and *f*(rACC)=2.81 +/− 0.28 Hz, *P*>0.05/3, Bonferroni corrected *t*-test, measured when the cards appeared on the table at the beginning of each round, *n*=125, 95 and 138, Supplementary Fig. 4). No significant difference in firing rate was observed in response to the low-level difference in the individual card decks (suite/colour) in any of the three brain areas (*P*>0.05/3, Bonferroni corrected *t*-test, *n*=125, 95 and 138, Supplementary Fig. 4).

**Figure 2:**
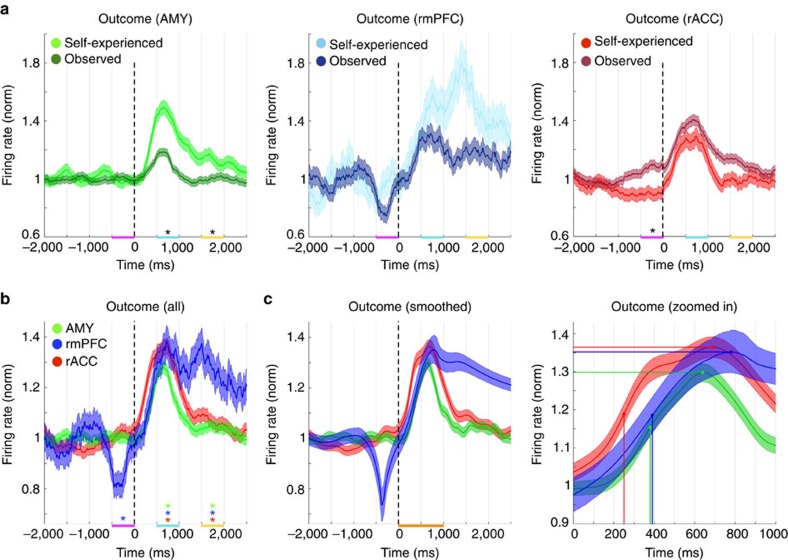
Comparing response envelopes in outcome responsive neurons. (**a**) Peristimulus time histograms (300 ms bin width, 10 ms step size) were calculated across selected units for self-experienced (light colours, mean +/− s.e. of the mean, s.e.m.) and observed trials (dark colours; balanced for high win/loss trials and low win/loss trials, respectively) and the mean firing rates in the two trial types were compared with each other in three different time intervals: choice (magenta), early response (cyan) and late response (yellow; *P<0.05/3, *t*-test, *n*_AMY_=1,920 and 3,840, *n*_rmPFC_=540 and 1,080, *n*_rACC_=1,440 and 2,880). (**b**) Combining the self-experienced and observed trials revealed a significant decrease in the rmPFC firing rate during choice and a significant increase in all three brain areas during the early and late response periods (*P<0.05/9, *t*-test compared with −3,000 to −1,000 ms, *n*_AMY_=5,760, *n*_rmPFC_=1,620, *n*_rACC_=4,320). (**c**) The same data as in **b** smoothed and plotted with the bootstrapped 95% c.i. further emphasized the sharp hiatus in the rmPFC neurons' firing rate at −370 ms (half minimum onset at −470 ms and offset at −230 ms, left panel). The response in the rACC was significantly earlier (half-maximum, vertical lines) than in both the AMY and the rmPFC and higher than in the AMY (peak, horizontal lines; right panel, zoomed in on orange highlight in left panel).

To initially compare the mean neuronal response profiles across the three brain areas, both before and after outcome, we selected only units, which showed a significant increase in their mean firing rate across all card game trials at outcome, independent of the trial type or outcome (self-experienced/observed and win/lose respectively). For this comparison we used a conservative response criterion based on the h-coefficient^14^, which returned 32 units in the AMY, 9 in the rmPFC, and 24 in the rACC (5,760, 1,620 and 4,320 trials, respectively), analyzing three time periods: the choice period (−500–0 ms), an early response period (500–1,000 ms), and a late response period (1,500–2,000 ms, at *t*=0 ms the outcome was revealed). In the AMY we recorded a higher mean firing rate during self-experienced trials compared with observed trials during the early response period (*P*=0.002<0.05/3, Bonferroni corrected *t*-test, *n*=1,920 and 3,840) and the late response period (*P*=0.003<0.05/3 Bonferroni corrected *t*-test, *n*=1,920 and 3,840). In the rmPFC, we found the same numerical difference, but the effect was not significant (*P*>0.05/3, Bonferroni corrected *t*-test, *n*=540 and 1,080). Conversely, in the rACC the response properties were reversed, displaying a higher firing rate during observed trials as compared to self-experienced trials. These rACC firing rates were found to be significantly different between the two trial types during the choice period, before the outcome was revealed (*P*=0.006<0.05/3, Bonferroni corrected *t*-test, *n*=1,440 and 2,880; Fig. 2a).

To further compare the mean response envelopes across the three brain areas we analyzed all trials combined (self-experienced and observed) and compared the mean firing rate during the three time periods to a pre-choice period serving as baseline (−3,000 to −1,000 ms). This analysis revealed a significant, sharp cessation of activity in the rmPFC, shortly before the outcome was revealed (*P*=0.0009<0.05/9, Bonferroni corrected *t*-test, *n* =1,620; Fig. 2b). 10,000 bootstrapped smoothed^15^ mean response envelopes further emphasized the sharp cessation of firing during the choice period in the rmPFC and were used to measure response onset times (half-maximum) and response amplitudes (Fig. 2c). The response onset in the rACC (249.986 +/− 30 ms, 95% c.i.) was significantly earlier than in the AMY (380.325 +/− 35 ms, *P*=<10^−5^<0.05/3, Bonferroni corrected *t*-test, *n*=10,000) and the rmPFC (385.19 +/− 85 ms, *P*=0.0026<0.05/6, Bonferroni corrected *t*-test, *n*=10,000), while no difference in onset time was observed between the AMY and the rmPFC (*P*=0.5>0.05/6, Bonferroni corrected *t*-test, *n*=10,000). The amplitude of the responses was higher in the rACC than in the AMY (*P*=0.001<0.05/6, Bonferroni corrected *t*-test, *n*=10,000) but not significantly different between the rACC and the rmPFC or between the rmPFC and the AMY (*P*>0.05/6, Bonferroni corrected *t*-test, *n*=10,000).

### Outcome encoding

**Figure 3:**
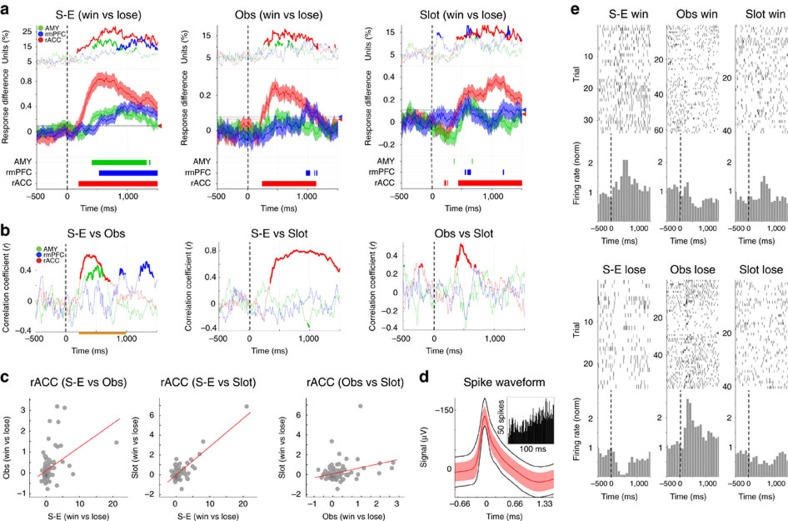
Outcome encoding. (**a**) The mean response difference between winning and losing (at *t*=0 ms) in self-experienced trials in the AMY, rmPFC and rACC (*n*=125, 95 and 138, respectively). Top panels: percentage of neurons with values outside of the 95-percentile of values recorded during the 1 s immediately before outcome (bold lines show percentages which are higher than expected based on a binomial test with *α*=0.01). Middle panels: the mean (+/− s.e.m.) response difference values (arrowheads and horizontal lines mark the upper end of the 95% c.i. of 10,000 bootstrapped means during the 1 s before outcome). Bottom panels: time points where the mean response difference values were significantly different from those before outcome (*P*<0.01/3, *t*-test, *n*_AMY_=125, *n*_rmPFC_=95, *n*_rACC_=138). (**b**) Pearson correlation coefficients between the individually plotted mean response difference values from **a**, bold traces highlight a significant correlation between the two variables (*P*<0.01/3, *n*_AMY_=125, *n*_rmPFC_=95, *n*_rACC_=138). (**c**) The same analysis as in **b** in the rACC but with values averaged over the time window highlighted there in orange. Every data point represents the mean response difference between winning and losing in a single rACC neuron, in all three comparisons we found a significant Pearson correlation (*p* (S-E versus Obs)<10^−4^<0.01, *r*(S-E versus Obs)=0.425; *p*(S-E versus Slot)<10^−4^<0.01, *r*(S-E versus Slot)=0.812; and *p*(Obs versus Slot)<10^−4^<0.01, *r* (Obs versus Slot)=0.311, *n*=138). (**d**) Waveform of all spikes recorded from an rACC neuron (*n*=10,828, mean +/− s.e.m in red, middle 95% of values in black, insert: interspike interval frequency plot). (**e**) For the same neuron as in **d** raster plots and peristimulus time histograms of wins (top) and losses (bottom) in self-experienced (left), observed (middle), and slot machine trials (right). This example neuron not only responds differentially to winning and losing in all three trial types, but notably does so inversely for observed outcomes compared with self-experienced and slot machine outcomes.

After finding specific differences in response envelopes and onset times between the three brain areas, we investigated the three complete neuronal populations' general response properties to winning versus losing. For this analysis, we measured the absolute mean difference in each individual neuron's firing rate between winning and losing trials (subtracting the mean differences before the outcome was revealed, −1,500–0 ms, cf. Fig. 2a right panel). In self-experienced trials this mean response difference increased in all three brain areas after the outcome was revealed (*t*=0 ms, Fig. 3a). However, only the neuronal population in the rACC also showed an increase of the mean response difference after outcome in both observed and slot machine trials, while in the AMY and rmPFC this effect was only very weak or absent (repeated *t*-tests and mean response difference higher than the 95% of 10,000 bootstrapped means calculated over the pre-response period, Fig. 3a). We then asked whether it is the same rACC neurons that encode outcome across all three different trial types. If this were the case, we would expect the rACC population's mean response difference values to be correlated between any given pairing of trial types (for example, self-experienced versus observed trials). While we found such a correlation in all three brain areas between self-experienced and observed outcomes, only in the rACC did we find that the mean response difference values were indeed correlated across all three trial type pairings (self-experienced versus observed, self-experienced versus slot machine, and observed versus slot machine; *P*<0.01, Pearson correlation over time and also during a predefined response period, *n*=138 neurons; Fig. 3b,c and Supplementary Fig. 5). These results indicate that not only do rACC neurons encode winning versus losing in all three trial types, but also that a subset of rACC neurons individually encoded all three outcome types; self-experienced outcomes, observed outcomes, and even outcomes in the slot machine trials, an entirely different win/lose task (for an example see Supplementary Fig. 6; notably, in some cases a reversal of the response direction between trial types could also be observed. For example the unit in Fig. 3d,e displayed what might be termed a *shadenfreude* response, increasing its firing rate for self-experienced wins and observed losses and decreasing its firing rate for self-experienced losses and observed wins).

### Amount encoding

**Figure 4:**
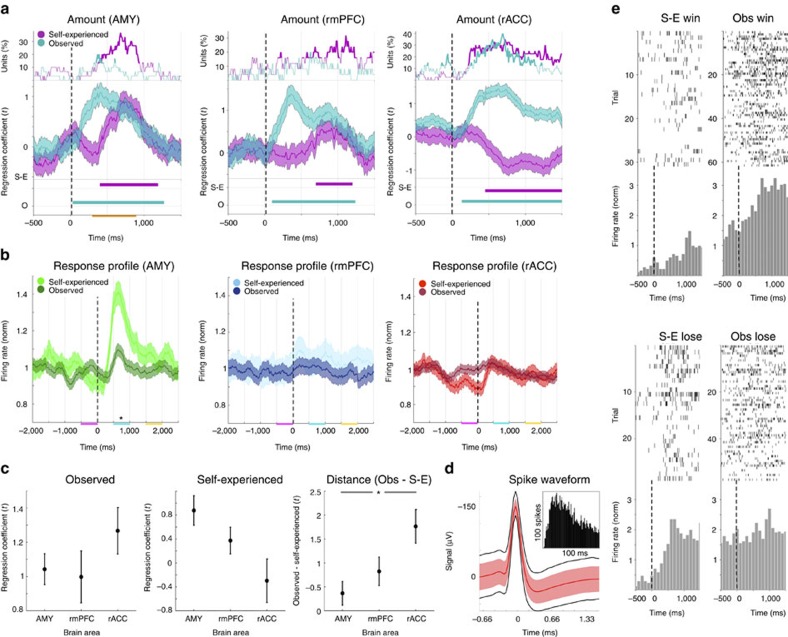
Amount encoding. In this analysis only neurons showing a positive regression of their firing rate to the observed amount were included (*n*=30, 25 and 40 in the AMY (left), rmPFC (middle) and rACC (right), respectively). (**a**) Top panels: same as in Fig. 3. Middle panels: the mean (+/− s.e.m.) *t*-statistic of the regression coefficients of the firing rates to self-experienced amounts and observed amounts (revealed at *t*=0 ms). Bottom panels: time points after outcome with mean regression coefficient *t*-statistic values, for the sum of which a cluster statistical analysis across 10,000 label shuffled datasets was significant (*α*<0.01). We note that an increase in the regression coefficient for observed amounts after outcome is caused by the implicit selection bias; for self-experienced amounts, however, no selection bias was present. (**b**) The peristimulus time histograms of the selected populations of units were analyzed in the same way as in Fig. 2a revealing a significant difference in the AMY response amplitudes between self-experienced and observed trials (*P<0.05/3, *n*=1,800 and 3,600). (**c**) The mean values of the regression coefficients in **a** during the outcome period (300–900 ms, orange line in **a**). *Post-hoc* testing revealed a significant difference in the distance between observed and self-experienced values between the AMY and the rACC (*P<0.05/3, *t*-test, *n*=30 and 40 right panel). (**d**) Spike waveform of an example neuron presented as in Fig. 3d (*n*=10,536). (**e**) Raster plots and peristimulus time histograms for the same neuron as in **d** showing higher firing rates for self-experienced losses than for self-experienced wins and higher firing rates for observed wins than for observed losses reflecting the findings in **a**, left panel.

In the unsigned and averaged population analysis of mean response difference values, more subtle, directional coding or the encoding of task variables within subpopulations of neurons may remain unobserved. We therefore additionally investigated, whether a subpopulation of neurons, specifically selected for directionally encoding the amount won or lost in observed trials (−$100, −$10, +$10 or +$100), also encoded that same parameter in self-experienced trials. In this analysis only neurons were included, whose firing rate increased as the observed amounts increased in at least one time point after the observed outcome was revealed (300–900 ms, *P*<0.05/2, Bonferroni corrected for positive and negative regression coefficients; *n*_AMY_=30, *n*_rmPFC_=25, *n*_rACC_=40). In the AMY and rmPFC this selected subpopulation of neurons on average also positively encoded self-experienced amounts, increasing their firing rate as the subject gained higher amounts (*P*<0.01, cluster statistical analysis over 10,000 equivalent but label shuffled datasets, Fig. 4a; the same selected subpopulation of neurons in the AMY also fired significantly higher in response to self-experienced outcomes than to observed outcomes, *P*<10^−5^<0.05/3, Bonferroni corrected *t*-test, *n*=1,800 and 3,600 trials, Fig. 4b). In the rACC, however, the selected subpopulation of neurons on average encoded self-experienced amounts with the opposite, negative sign, decreasing their firing rate as the subject's gains increased (*P*<0.01, cluster statistical analysis over 10,000 equivalent but label shuffled datasets, Fig. 4a; Supplementary Fig. 7).

We compared this amount encoding across the three brain areas using the mean *t*-statistics of the regression coefficients for amount won or lost over the whole response period (300–900 ms). In this comparison, we found no significant effect for observed amount encoding across the three areas (F (2, 92)=1.315, *P*=0.274>0.05, ANOVA, *n*=95 neurons; Fig. 4c), but we did measure an effect for self-experienced amount encoding (F(2, 92)=5.484, *P*=0.006<0.05, ANOVA, *n*=95). Taken together, the distance between self-experienced and observed values forms a measure of the asymmetry between self versus observed amount encoding. This measure also showed a significant effect across the three brain areas (F(2, 92)=5.484, *P*=0.006<0.05, ANOVA, *n*=95). *Post-hoc* comparisons of this distance measure revealed no significant difference between the AMY and the rmPFC (*P*=0.238, Bonferroni corrected *t*-test, *n*=30 and 25), nor between the rmPFC and the rACC (*P*=0.067, Bonferroni corrected *t*-test, *n*=25 and 40), but a significantly larger distance was measured in the rACC than in the AMY (*P*=0.003<0.05/3, Bonferroni corrected *t*-test, *n*=40 and 30). These results suggest that the difference in amount encoding between the three brain areas was driven by differential encoding for self-experienced and observed outcomes primarily between AMY and rACC, reflecting the observation that in these AMY units there was little difference between amount encoding for self and other, while amount was encoded with opposite signs for self-experienced and observed outcomes in rACC (for an example of a single rACC neuron displaying this type of encoding see Fig. 4d,e; for localization of the neurons selected in this analysis see Supplementary Fig. 8a; when instead selecting for self-experienced amount encoding neurons, the same directionality was observed but only reached significance in the AMY, *P*<0.01, cluster statistical analysis over 10,000 equivalent but label shuffled datasets, Supplementary Fig. 8b).

### Encoding of observational learning parameters

Having found evidence of observed outcome and amount encoding, we investigated if these effects may contribute to the encoding of observational PEs. We therefore tested if neurons not only encoded how rewarding an observed event was (outcome amount) but also how rewarding it was expected to be (expected value). In particular, we tested specific predictions of algorithms originally developed in artificial intelligence known as temporal difference learning models^16^. The prediction error term from these learning algorithms has been shown to closely resemble the activity of a subpopulation of primate dopamine neurons during self-experienced reinforcement learning^2^,^5^,^17^. Beyond the PE when the outcome is revealed, temporal difference models additionally postulate that a PE signal should occur at the earliest predictive event, signalling the difference between the new expected value and the immediately preceding expected value. In observed trials this occurs at the point at which the other player's choice is revealed (card highlighted, Fig. 1b), and, in our task setting, is approximated by the expected value of the observed choice. We therefore tested if neurons encoded the following tripartite coding scheme during observed trials: a positive expected value signal before the outcome was revealed (a positive correlation of their firing rate to the expected value at the point of choice); a positive signal of the amount won or lost by the observed player (a positive correlation of their firing rate to the amount); and a negative expected value signal after the outcome was revealed (a negative correlation of their firing rate to the expected value but after the outcome was revealed), with the combination of and constituting the full PE signal—the amount obtained minus the expected value. We selected neurons based on prediction by only including units that showed a positive effect of the expected value at choice (*P*<0.05/2, Bonferroni corrected for positive and negative regression coefficients, in at least one time point during the choice period of −900 to –300 ms; *n*_AMY_=14, *n*_rmPFC_=9, *n*_rACC_=22), and then tested for , a positive effect of amount and , a negative effect of the expected value, after the outcome was revealed. Note that selection of these units during choice means that there is no selection bias for statistical tests performed during outcome.

**Figure 5:**
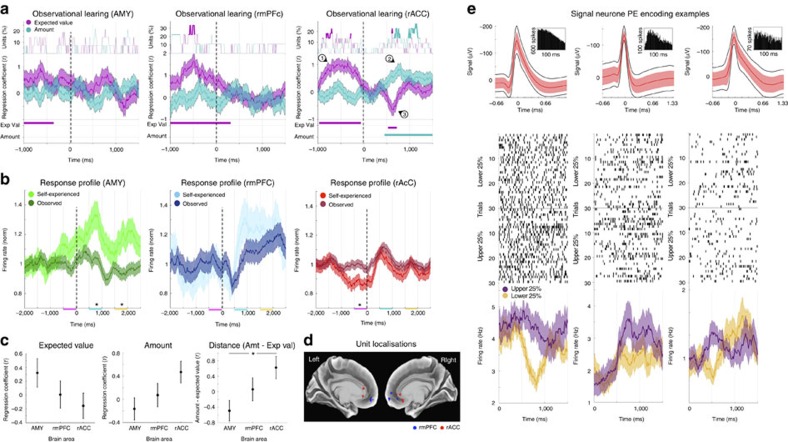
Observational learning in the rACC. This analysis only included neurons with a positive regression of their firing rate to the expected value during observed choice (−900 to −300 ms). The data is presented in the same way as in Fig. 4. (**a**) In the AMY (left, *n*=14) and rmPFC (middle, *n*=9) the selected neurons did not show any significant PE encoding after the outcome was revealed. However, in the rACC (right, *n*=22), besides the selected-for predictive encoding of the expected value (), neurons additionally encoded the amount positively () and the expected value negatively () at outcome, as postulated by formal learning theory. (**b**) The selected neurons in the AMY fired higher during self-experienced trials than during observed trials in the early and late response period, while in the rACC the firing rate during self-experienced trials was reduced significantly during the choice period (*P<0.05/3, *t*-test, *n*=1,320 and 2,640). (**c**) *Post-hoc* testing revealed a significant difference in the distance between the amount term and the expected value term between the AMY and the rACC (*P<0.05/3, *t*-test, *n*=14 and 22, right panel). (**d**) Localization of the recording sites of the neurons selected in this analysis (MNI space, Supplementary Table 1). (**e**) Three examples of individual neurons from the rACC subpopulation selected in this analysis presented as in Fig. 3d,e but showing the mean (+/− s.e.m) for the upper and lower quartile (25%) of trials ordered according to their PE values. All three units show a higher firing rate for the upper quartile than the lower quartile after the outcome was revealed (at *t*=0 ms; the middle panel shows the same unit as in Fig. 4).

Using these criteria, the selected neurons in the AMY and rmPFC did not show a significant observational PE signal (compared with 10,000 equivalent but label shuffled datasets, Fig. 5a; as above, these selected AMY neurons also responded with a higher firing rate during self-experienced trials than during observed trials during the early and late response periods, *P*=0.002 and *P*=0.003<0.05/3, Bonferroni corrected *t*-tests, *n*=840 and 1,680 respectively, Fig. 5b). In the rACC, however, during observed trials the selected subpopulation of units did encode a positive PE by encoding the amount positively (*P*<0.01) and the expected value negatively at outcome (*P*<0.01, cluster statistical analysis over 10,000 equivalent but label shuffled datasets, Fig. 5a; Supplementary Fig. 9; the selected rACC neurons encoding this observational PE also significantly decreased their firing rate during the self-experienced choice period, before the outcome was revealed, *P*=0.006<0.05/3, Bonferroni corrected *t*-test, *n*=1,320 and 2,640, Fig. 5b; we did not observe any significant PE encoding when selecting units in the same way but for self-experienced trials, although the encoding profile in rmPFC was suggestive; Supplementary Fig. 10). Comparing the mean *t*-statistics of the regression coefficients over the whole response period (300–900 ms), we found no difference between the three brain areas for observed expected value encoding (F (2, 42)=1.561, *P*=0.222>0.05, ANOVA, *n*=45) or observed amount encoding (F (2, 42)=2.919, *P*=0.065>0.05, ANOVA, *n*=45; Fig. 5c) on their own. We did, however, measure a significant difference between the observational prediction error effect, defined as the amount effect minus the expected value effect (the distance between the two), across the three brain areas (F(2, 42)=3.964, *P*=0.026<0.05, ANOVA, *n*=45). *Post-hoc t*-test comparisons revealed no significant difference in this PE term between the AMY and the rmPFC (*P*=0.189, *n*=14 and 9) or between the rmPFC and the rACC (*P*=0.26, *n*=9 and 22), but a significant difference was found between the AMY and the rACC (*P*=0.011<0.05/3, *n*=14 and 22).

The rACC subpopulation of neurons encoding observational learning parameters (22 out of 138 rACC units) was localized predominantly in the rostral gyral subdivision of the cingulate cortex (Fig. 5d). The analysis of the regression coefficients (Fig. 5a) demonstrates a linear relationship between the firing rate and the prediction error. In a more conservative analysis, in which however this linear relationship is lost, we additionally investigated to what extent we could still observe the same coding scheme by simply comparing the firing rates during high and low PE value trials with each other. In this test, 15 of the original 22 selected neurons still showed the same effect (time points with higher firing rate and non-overlapping s.e.m. in the upper as compared to the lower quartile of PE trials during the response period; for example Fig. 5e, the neuron in the middle panel being the same unit as in Fig. 4). While the number of units selected in this less sensitive analysis is relatively small (15 out of 138 rACC units) it is still significantly higher than expected by chance (*P*=0.0015<0.01 in a binomial test).

To further characterize the observational PE effect in this rACC subpopulation, we carried out two additional analyses. First, we binned each neuron's firing rate into terciles sorted by the model-derived PE term and computed the mean firing rate during the response period across all selected neurons (300–900 ms). This analysis revealed monotonic positive encoding of the observational PE term (Supplementary Fig. 11a). This finding confirms a positive monotonic relationship between the observational PE term and average firing rate in this subpopulation; however, we do not have the statistical power to make any claims about the nature of the monotonic function (for example, linear or nonlinear). Second, we performed a model-free analysis that leveraged the structure of the card game task. Although win/loss outcomes were stochastic, and these outcomes were only predicted with a 0.7/0.3 probability, it should nevertheless be the case that on average the same win outcomes of the same amount and player should be more predictable late within a game than immediately following the transition to a new game. To test this prediction, which does not rely on our computational learning model, we compared early wins (during the first two rounds of a game) to late wins (during the final two rounds) for all three players separately (self-experienced and observed Obs_1_ and Obs_2_, see Fig. 1a). In particular, for each neuron we subtracted the mean firing rate for early wins from that for late wins for both high (+$100) and low (+$10) amounts individually, allowing us to compare identical win outcomes, which were either more (late) and less (early) expected. This model-free analysis revealed a trend towards a higher firing rate when the identical win outcome occurred early in a game compared with late in a game for observed trials (*P*=0.05), but not for self-experienced trials (*P*=0.46; paired *t*-test between observed and self-experienced outcomes: *P*=0.06, Supplementary Fig. 11b). This result provides convergent evidence that this subpopulation of rACC neurons encodes observational, but not self-experienced, PEs. We note however, that by construction this analysis will be less sensitive than the model-based regression analysis described above because it includes some late trials where the prediction error is larger than in early trials due to the stochastic generation of outcomes and the relatively uncertain true reward generating probabilities (0.7/0.3) implemented in the task (Fig. 1d).

## Discussion

We have characterized the responses of single neurons in human AMY, rmPFC, and rACC during self-experienced and observational learning for the first time. Our findings show a diversity of responses across all three brain areas examined, but also highlight selectivity between areas. Although neurons in all three brain areas showed clear encoding of outcomes and amount during self-experienced trials, neurons in rACC were most strongly recruited when observing the other players. The subpopulation of responses in AMY and rmPFC that did encode amount for observed outcomes encoded amount in the same direction for self-experienced outcomes, whereas the responses tended to be encoded in opposing directions in rACC. Further analyses demonstrated that the firing rate of a subpopulation of rACC neurons reflected a signature of a prediction error predicted by formal learning theory but during observational trials—a positive signal encoding the expected value of a choice when it was observed (the earliest predictive event for the outcome) and a prediction error after the outcome was delivered. By dividing the prediction error term into its component parts, we could further show that this signal did in fact reflect a prediction error—the outcome amount minus the expected value, indicating that these responses were not simply driven by encoding of the amount alone. Notably, this subpopulation did not encode self-experienced prediction errors, and this tripartite scheme was not observed in AMY or rmPFC, demonstrating the functional and anatomical selectivity of this result.

Our findings extend and complement the results of past studies on social decision making and learning in humans and monkeys. In particular, functional magnetic resonance imaging studies have reported various signals related to observational learning in regions including the striatum, the ventromedial PFC and, notably, the ACC gyrus^12^,^18^,^19^,^20^,^21^,^22^, providing evidence of the involved structures and computations but not the actual underlying neuronal mechanisms, and a macaque lesion study previously demonstrated a causal role for the ACC gyral subdivision in attention to social stimuli^23^. The few studies that have actually recorded single-neuron activity during social interaction reported cells in subdivisions of medial frontal cortex in monkeys and, in one case, humans responding to observed binary actions, including response errors^24^,^25^, observed (binary) reward omission^25^, and even donated rewards^26^. While these studies implicate single neurons in medial frontal cortex, in general, and ACC gyrus, in particular, in the processing of socially relevant signals, our study goes beyond these findings by providing insight into the actual single-cell computations at work when learning through observation.

Past studies recording single units in human ACC^27^,^28^,^29^ have instead focused on recordings localized in the more caudal and dorsal aspect of the cingulate cortex, typically in or around the cingulate sulcus in the rostral or caudal cingulate zone^30^. These studies have emphasized a role for dorsal ACC neurons in the integration of rewards and actions, monitoring of general cognitive and emotional demands, and behavioural adaptation^27^,^28^,^29^, consistent with theories on dorsal ACC^31^. In addition, self-experienced reward prediction errors have been reported in this dorsal anterior cingulate sulcal subdivision using non-invasive measures in humans^32^ and in macaque single units^7^. By contrast, the majority of neurons described in this study are located considerably more rostral and ventral within cingulate cortex, mostly in a peri-genual and gyral region^30^ (Figs 1e and 5d). This subdivision of the ACC has been more closely linked to social attention, learning, and empathy^23^,^33^,^34^, although there have been very few single unit recording studies targeting this area, with one notable exception^26^. Interestingly, while we found observational PE encoding in this subpopulation, we failed to find self-experienced PE encoding. This difference may be explained by the previously described functional selectivity between more dorsal, sulcal and ventral, gyral subdivisions within ACC^12^,^23^,^33^. However, since there were half as many self-experienced than observed trials, and their temporal order was fixed, the null finding on self-experienced trials may be underpowered and should be interpreted with caution.

By comparing single neurons in rACC, rmPFC, and AMY in the same task for the first time in any species, we could further demonstrate the selectivity of rACC coding during observational learning, as well as elucidate commonalities and differences between neuronal responses in these three brain areas (Figs 2, 3, 4, 5). For example, correlation analyses of effect size measurements between winning and losing outcomes (absolute mean difference in firing rate) over different trial types showed encoding of self-experienced and observed outcomes within individual single neurons in all three areas. However, only neurons in rACC encoded these two outcomes as well as outcomes in the slot machine task. It is important to note that AMY and rmPFC neurons were engaged in the slot machine task (*y* axis in Supplementary Fig. 5, middle and right panels), but these were not the same units as those, which responded during the card game task. We also observed that, for self-experienced and observed trials, neurons in the AMY and the rmPFC encoded the amount won or lost positively, reflecting recently reported single-neuron findings in monkey AMY^35^, while rACC neurons showed opposing coding schemes for these two trial types. Collectively, these findings suggests a more general and at the same time more specific engagement of single rACC neurons in the processing of different sources of information and across different behavioural contexts relevant to observational learning.

Learning through observation plays a crucial role in human development, everyday life, and society^11^. We designed a task to measure observational learning computations in single neurons in humans. The recorded data allowed us to show that the activity in a subpopulation of peri-genual ACC neurons located primarily in the gyrus encodes observational predictions and PEs, consistent with formal learning theory and in particular temporal difference learning algorithms, but heretofore never demonstrated empirically in any species. These findings establish for the first time in humans, a direct relationship between computation in individual neurons and its function at the level of behaviour.

## Methods

### Data acquisition

The data presented in the current study were recorded in 31 sessions across 10 patients suffering from pharmacologically intractable epilepsy (mean age 36.9 +/− 3.6 years, six women, no significant difference in age between genders, *P*=0.292>0.05, *n*=6 and 4). The study protocols were approved by UCLA's Institutional Review Board and informed consent was obtained from all participating subjects. As part of their clinical diagnostic procedure these patients were implanted with chronic depth electrodes for 1–3 weeks to localize seizure foci for possible surgical resection^13^. Through the lumen of the clinical electrodes nine Pt/Ir microwires were inserted into the tissue, eight active recording channels and one reference. The differential signal from the microwires was amplified and sampled at 30 kHz using a 128-channel BlackRock recording system. Spikes in the continuous data were detected and sorted using wavelets and super-paramagnetic clustering^36^. Only single-units were included in the data presented here. Single units were classified and distinguished from each other visually based on the following four criteria: the spike shape and its variance; the interspike interval distribution; the presence of a refractory period (<3–5% of all interspike intervals within a unit had to be shorter than 3 ms, Supplementary Fig. 3a); and the ratio between the spike peak value and the noise level (the number of s.d. of the noise, that the peak of the mean spike was above the mean of the noise, Supplementary Fig. 3b). In cases where more than one unit was recorded within an electrode, extra care was taken to ensure that the clustered units were qualitatively substantially different from each other in regard to points 1 and 2 above (for examples, see Supplementary Fig. 3c). All studies conformed to the guidelines of the Medical Institutional Review Board at UCLA.

### Anatomical localization

The electrode locations were based exclusively on clinical criteria. In the current study data from the AMY, the rmPFC (putative Brodmann's areas 10m, 10r), and the rACC (areas 24 and dorsal 32) are presented^37^. To anatomically localize single-unit recording sites we registered computerized tomography images acquired post-implantation to high-resolution T1-weighted magnetic resonance imaging data acquired pre-implantation using FLIRT, part of FMRIB's Software Library (www.fmrib.ox.ac.uk/fsl^38^). Microwire localization for each subject was performed manually by hand-drawing masks separately for each region using the FSLview tool. Amygdala units were defined by the neurosurgeon pre-implantation, and then verified on each subject's high-resolution T1-images. rACC units were defined to include units localized in anterior cingulate gyrus, anterior cingulate sulcus, and dorsal paracingulate gyrus. rmPFC units were defined as those localized rostral and/or ventral to the rACC units (Fig. 1e). The T1-weighted images were then affine-registered into standard Montreal Neurological Institute space, and subject-specific localization masks were transformed into standard space using the same transformation matrix. To visualize units on the medial cortical surface, we projected masks onto the Caret Conte69 human surface-based atlas available in the Human Connectome Toolbox (humanconnectome.org^39^).

### Experimental paradigm

Subjects executed the card game task followed by the slot machine task on a laptop computer, while sitting up in their hospital bed. The other two, observed players were introduced to the subjects by asking them to read individual short biographies and imagining them to be physically present and actually playing cards with them. Due to clinical restrictions they were not real people present in the room, but instead their choices were played out by the computer. The subjects were made aware of these restrictions. They were additionally (wrongfully) informed that the other players' choices were pre-recorded and asked to imagine playing against these real people. After conclusion of all experiments, the subjects were debriefed that the observed players were in fact made up virtual agents and that their behaviour was deliberately designed by the experimenters as to maximize amount and PE variance over trials.

In an individual trial the current player (the subject or one of the two observed players, Fig. 1a) had to draw a card by choosing one of two decks (the subjects made their choice with the left or right arrow key). On a player's choice, the chosen card was highlighted for 1 s before the outcome was revealed for 3 s (Fig. 1b). We recorded 180 trials per session (60 self-experienced trials and 120 observed trials). The subjects were aware that no real money would be paid out (their total winnings were always visible onscreen while the other players' total winnings remained unknown). Additional motivation was provided by designing the experimental paradigm as an appealing computer game including a dedicated hall of fame across all anonymized patients. Patients reported a strong motivational effect resulting from competing in this hall of fame. They started playing the slot machine with the higher amount won during two sets of six card games played immediately prior. In the slot machine game winning doubled their total earnings and losing halved them, however, they could never go below $ 0. Subjects played the slot machine until they had played for at least 5 min and decided to stop (on average 79.6 +/− 8.2 trials were played out). All subjects easily followed these instructions with the exception of one individual, who was therefore not included in this study.

### Neuronal data analysis

Mean firing rates over time (Figs 2, 3, 4, 5), response envelopes (Fig. 2b), measures of absolute mean response difference (Fig. 3a), Pearson correlations of these with each other (Fig. 3b), and regression coefficients in the Bayesian model (Figs 4 and 5), were all measured in a moving average window of 300 ms with a step size of 10 ms. To be included in the analysis of the response envelopes, shown in Fig. 2, neurons had to fulfil at least one of the following three conservative selection criteria: (1) a *P*-value<0.01 in a one-tailed paired *t*-test between pre-outcome (−900 to –300 ms) and post-outcome (300–900 ms); or (2) a *P*-value<0.05 and an *h*-coefficient^14^>=1; or (3) an *h*-coefficient ≥1.5. To test whether single neurons encoded key learning parameters, we defined a general linear model to regress against the firing rate of each identified single-unit in the rACC, rmPFC, and AMY from −1,000 to 1,500 ms around the outcome (*t*=0 ms). This model consisted of nine explanatory variables (or regressors): (1) main effect of self-experience trials: 60 trial events where the patient observed the outcome of their own choice; (2) self-experience modulated by the expected value (Eq. 7; Supplementary Methods) of the deck the subject selected; (3) self-experience modulated by the amount the subject won or lost at feedback; (4) main effect of the first observed player (Obs_1_): 60 trial events when the patient observed the outcome of Obs_1_'s choice; (5) Obs_1_ modulated by the subject's expected value of the deck Obs_1_ selected; (6) Obs_1_ modulated by the amount won or lost by Obs_1_; (7) main effect of the second observed player (Obs_2_): 60 trial events when the patient observed the outcome of Obs_2_'s choice; (8) Obs_2_ modulated by the subject's expected value of the deck Obs_2_ selected; (9) Obs_2_ modulated by the amount won or lost by Obs_2_. We then defined the following additional contrasts of parameter estimates between explanatory variables to define terms for observed trials: observed expected value=5+7; observed amount=6+8; observed prediction error=(6+8)−(5+7) (corresponding to the amount obtained minus the expected value, averaged over Obs_1_ and Obs_2_).

On the basis of formal learning theory and past studies on self-experienced reward learning^7^,^17^, we predicted that units selected for a positive effect of the observed expected value before outcome should also show a positive effect of observed amount and a negative effect of the observed expected value after the outcome was revealed (Fig. 5). To test for significant encoding of amount (Figs 4a and 5a) and the expected value (Fig. 5a), we performed cluster statistical analysis on the *t*-statistics of the regression coefficients resulting from these contrasts. For these analyses we calculated the mean *t*-statistics of the regression coefficients across the same number of neurons as in the original test set (the number of neurons returned by the selection criterion) 10,000 times. Each time random neurons were selected from all the units within the same brain area and within each of these randomly selected neurons one of 1,000 permutations was chosen, also at random, where in every permutation the trial labels were shuffled randomly. A fluctuation in the mean *t*-statistic value across time in the original test set was regarded as significant if the sum across consecutive values above or below an arbitrary threshold of +/− 0.2 lay outside of the 99-percentile of these values in the shuffled data (that is, *P*<0.01).

### Structure of the learning task

On each trial, subjects selected one of two decks {D1, D2} and observed one of two outcomes: a win (O_1_) or a loss (O_2_). Since, the subjects were informed that each stimulus was associated with exactly one outcome on each trial and these were inversely related, this single observation gave full information about the stimulus-reward contingencies on the current trial:


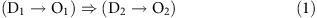


Hence, estimating the reward contingency for one deck *p*(D_1_→O_1_) is equivalent to estimating the full contingency structure. Let the true probability that D1 leads to O_1_ on trial *t*, *p*_*t*_(D_1_→O_1_), be denoted by *q*_*t*_. Then:


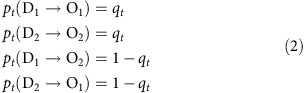


The true value of *q*_*t*_ was in fact 0.7 or 0.3, and these values were known by the subjects.

The subjects were instructed that the contingencies could reverse but were not told when; let the presence of a reversal on trial *t* be denoted by *J*_*t*_ such that


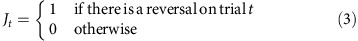


then


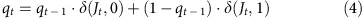


where *δ* denotes the Kroenecker delta function.

Subjects were instructed as to the probability of reversal; the contingencies reversed at the beginning of a new game (every 15 trials) with probability 0.5.

### Bayesian learning model

We constructed a normative Bayesian learning model that estimated the reward contingency *q*_*t*_ on each trial based on the history of observed outcomes and selected decks on trials up to and including trial *t*, denoted by ***y***_***1:t***_.

On each trial *t*, the posterior probability for each value of *q*_*t*_ was given using Bayes' rule:


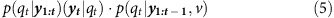


The likelihood 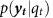 is simply *q*_*t*_.

The prior 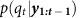 accounts for the possibility of a reversal *J.* The probability of a reversal *v*=*p*(*J*_*t*_=1) was modelled as fixed across trials but of unknown value. Hence, the prior 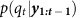 on trial *t* was obtained from the posterior on the previous trial by applying a transition function:


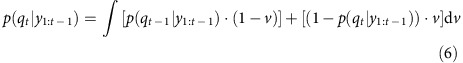


For simplicity, let r denote the mean of the belief distribution over rewards, given the past choice outcomes observed up to trial *t*: 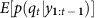; this is depicted by the magenta line in Fig. 1d. These normative predictions of reward probabilities constituted our estimate of a subject's expected value (EV) for a given deck 1:


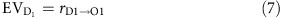


where 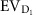 denotes the expected value for deck 1. It follows that the expected value of the alternative deck 2 is given by:


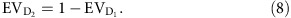


We assumed the subjects then selected between decks based on the following softmax distribution:


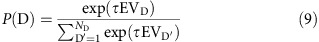


where *τ* is a subject-specific free parameter that reflects the sensitivity of deck choices to expected deck values and *N*_D_=2. We fitted *τ* to each individual subject's choices using standard nonlinear minimization procedures implemented in MATLAB 14a (Mathworks).

For behavioural regression analyses, we defined the choice entropy of the distribution over deck win probabilities as:


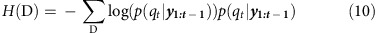


### Model-based behavioural analyses

To test whether the normative hierarchical Bayesian model we constructed captured meaningful features of subject behaviour, motivating its predictions of neuronal data, we tested whether two key terms—the expected value EV and the choice entropy CE, both derived from the model (without any fitting to behaviour), predicted trial-by-trial fluctuations in subjects' choices and choice times. In particular, we used logistic regression to test whether the likelihood of choosing the left deck on trial *t*, *p*_L,*t*_ (that is, the probability that the subject choice c_L,*t*_=1), was explained by the difference in EV between left and right decks *V*_L−R_ and the CE on trial *t*:


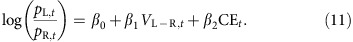


The coefficients represent changes in the natural logarithm of the odds of choosing the left deck (or equivalently the right deck since *p*_R*,t*_=1−*p*_L*,t*_). The model is linear in the log odds, but nonlinear in the probability of choice, which can be recovered by exponentiating both sides of the equation and solving for *p*_L,*t*_. We used the same model-derived terms *V*_L−R_ and CE to test whether they also explained trial-by-trial fluctuations in choice times, CT_*t*_, here using multiple linear regression:


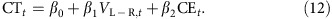


Positive coefficients indicate relative slowing in choice times.

To further test whether subjects learned from both the outcomes of their past choices and the outcomes of the observed players' choices, we performed an additional multiple linear regression analysis. Here, we sought to predict choices of the left deck, c_L,*t*_, as a function of past six outcomes (win or no win) when choosing the left (*r*_L,*t−j*_) or right deck (*r*_R,*t−j*_), both on self-experienced and observed outcomes:


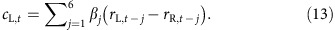


We then computed the mean over *β*_*j*_ on past trials corresponding to self-experienced outcomes (*t*−3 and *t*−6) and observed outcomes (*t*−1, *t*−2, *t*−4 and *t*−5) separately and computed corresponding *t*-statistics and *P*-values over the sample of subjects, reported in the main text and presented in Supplementary Fig. 2b.

To explicitly test to what extent the full prediction error term *δ*, defined as the reward amount obtained minus the chosen expected value, for self-experienced and observed outcomes, predicted subject choices, we used multiple linear regression but replaced past outcomes with the most recent prediction errors as the predictors for each player. This corresponded to trial *t*−3 for self-experienced outcomes and *t*−1 (Obs1) and *t*−2 (Obs2) for observed outcomes. Here we sought to predict choices of the left deck c_L*,t*_ as a function of the past three prediction errors when choosing the left deck *δ*_L,*t−j*_ and right deck *δ*_R,*t−j*_: 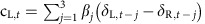.

*β*_*j*_ on trial *t*-3 corresponds to the weighting on the most recent self-experienced prediction error, and the mean over *β*_*j*_ on trials *t*−1 and *t*−2 corresponds to the combined weighting of the most recent observed prediction errors. We computed corresponding *t*-statistics and *P*-values over the sample of subjects, reported in the main text and presented in Supplementary Fig. 2c.

### Model-free prediction error analysis

To test whether more expected observed wins matched for amount won evoked a higher firing rate than less expected ones, we computed the group mean difference in firing rate (+/−s.e.m.) between early and late matched wins during the outcome response period (300–900 ms after outcome) for the subpopulation of single rACC neurons that showed a group observational PE response (Fig. 5). Differences were computed separately for self-experienced and observed trial outcomes. Early wins were defined as win outcomes that occurred in the first two of five trials for a particular player in a game of 15 trials, and these were averaged over the 12 games. Late wins were defined as win outcomes that occurred in the last two of five trials for a particular player in a game, and these were also averaged over the 12 games. The mean firing rate for low amount wins when they occurred in late trials was subtracted from low amount wins when they occurred in early trials and the same was performed for high amount wins. This means that identical win outcomes were compared when they should on average be less (early) or more (late) expected.

### Data availability

The data that support the findings of this study are available on request from the corresponding author (M.R.H.). The data are not publicly available due to privacy policies relating to these clinical recordings.

## Additional information

**How to cite this article:** Hill, M. R. *et al*. Observational learning computations in neurons of the human anterior cingulate cortex. *Nat. Commun.* 7:12722 doi: 10.1038/ncomms12722 (2016).

## Supplementary Material

Supplementary Information Supplementary Figures 1 - 11 and Supplementary Table 1
