# Supplementary material for: Observational learning computations in neurons of the human anterior cingulate cortex
Source: Nat Commun. 2016 Sep 6;7:12722. doi: 10.1038/ncomms12722 (PMC5025858; doi:10.1038/ncomms12722)
Supplement: Supplementary Information — Supplementary Figures 1 - 11 and Supplementary Table 1 [file ncomms12722-s1.pdf]

Supplementary materials

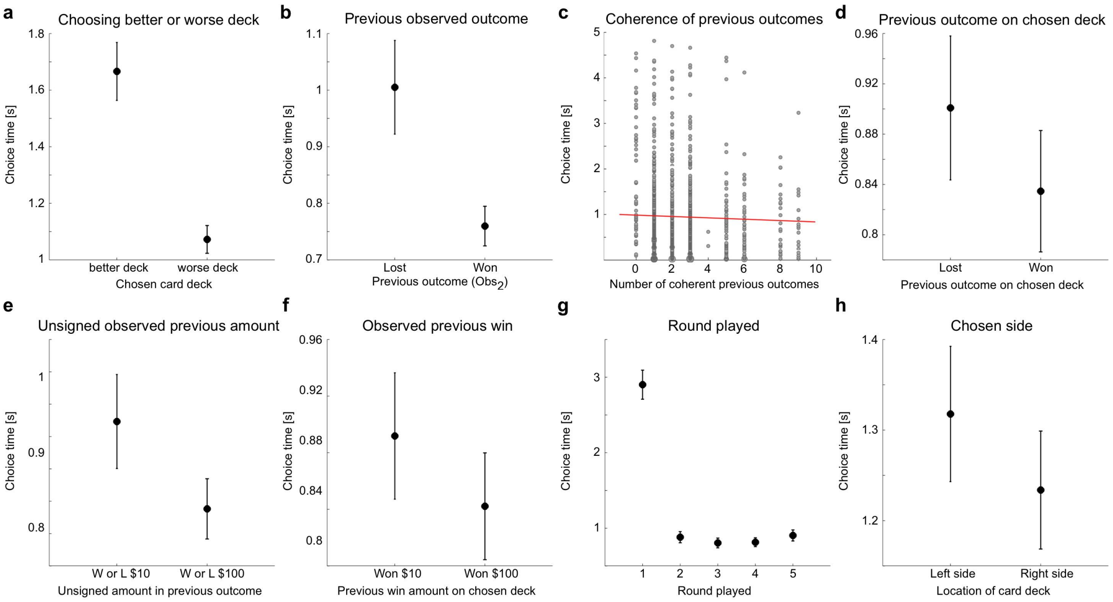

Supplementary Figure 1 | Choice times. The choice time was measured as the time it took for subjects to choose a card after the two decks appeared on screen and tested with a t-test, where not described otherwise. (**a**) Subjects were significantly slower when choosing the worse card deck as opposed to the better card deck ($p < 10^{-5}$, t-test, n = 1268 and 592,). (**b**) Choice times were significantly faster after observing a winning outcome during the previous player's trial (Obs_2, see Fig. 1a), than after observing a losing outcome (p = 0.003 < 0.05, t-test, n = 987 and 873). (**c**) A Spearman test revealed a significant negative correlation between the number of coherent previous trials and the choice time (p = 0.016 < 0.05, ϱ = -0.061, the marker size represents the number of identical data points ranging from 1 to 5, n = 1860). (**d**) No significant difference in choice time was observed between trials in which the previous outcome in the chosen deck was lost compared to those where it was won (p = 0.51 > 0.05, t-test, n = 894 and 517). (**e**) No significant difference in reaction time was observed between trials in which the unsigned previous outcome in the chosen deck was either low (won or lost $10) or high (won or lost $100; p = 0.114 > 0.05, 4 Outliers > 5 s.d. above the mean were discarded, n = 644 and 767). (**f**) The same analysis as in (e) but within winning outcomes only also revealed no significant difference in reaction time between trials in which the previous win in the chosen deck was low or high (p = 0.484 > 0.05, 7 outliers were discarded, n = 676 and 570). (**g**) Taking all 5 rounds into account an ANOVA revealed a significant effect of the round played on the reaction time (F(4, 1855) = 75.489, p < 10^(-5) < 0.05, n = 1860). Taking only round 2 - 5 into account no significant effect of the round played on the reaction time was observed (F(3, 1484) = 0.518, p = 0.67 > 0.05; 13 outliers were discarded, n = 1488). (**h**) No significant effect was found for the side, which the chosen card was lying on, on the reaction time (p = 0.401 > 0.05, 5 outliers were discarded, t-test, n = 966 and 894).

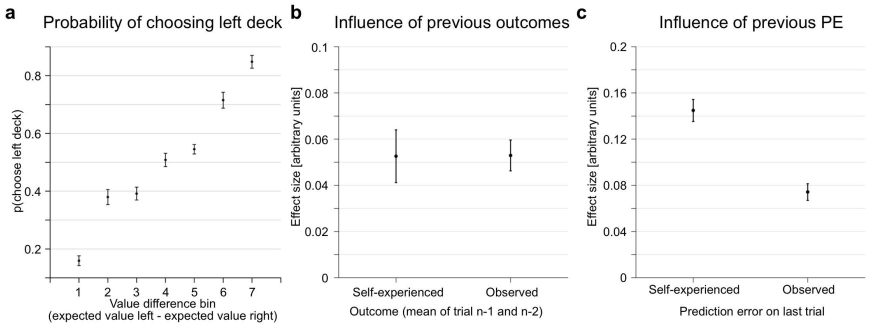

Supplementary Figure 2 | Model behavior. Relationship between choices and model-predicted expected values, past outcomes, and past prediction errors. (**a**) The mean (+/-s.e.m.) of the probability of selecting the left deck is plotted against seven equally distributed expected value difference bins (left expected value – right expected value). (**b**) Group mean +/-s.e.m of regression coefficients reflecting the influence of past binary win/loss outcomes for self-experienced and observed trials on the subjects' current choices. For both self-experienced and observed trials, regression coefficients from the past two trials were averaged together. Due to the task structure, this constitutes the mean of trials n-3 and n-6 for self-experienced trials and the mean of trials n-1, n-2, n-4, and n-5 for observed trials (see Fig. 1a). Significant positive effects show that subjects were more likely to select a specific card deck, if it had been selected and led to a win (compared to a loss), or the alternative card deck was selected and it led to a loss (compared to a win), in the previous two trials for both self-experienced and observed outcomes. (**c**) Subject group mean +/-s.e.m of regression coefficients reflecting the influence of past model-derived prediction errors from the most recent self-experienced trial (trial n-3) and the most recent observed trial for each observed player (mean of coefficients for observed PEs for Obs1 which occurred on trial n-1 and Obs2 which occurred on trial n-2).

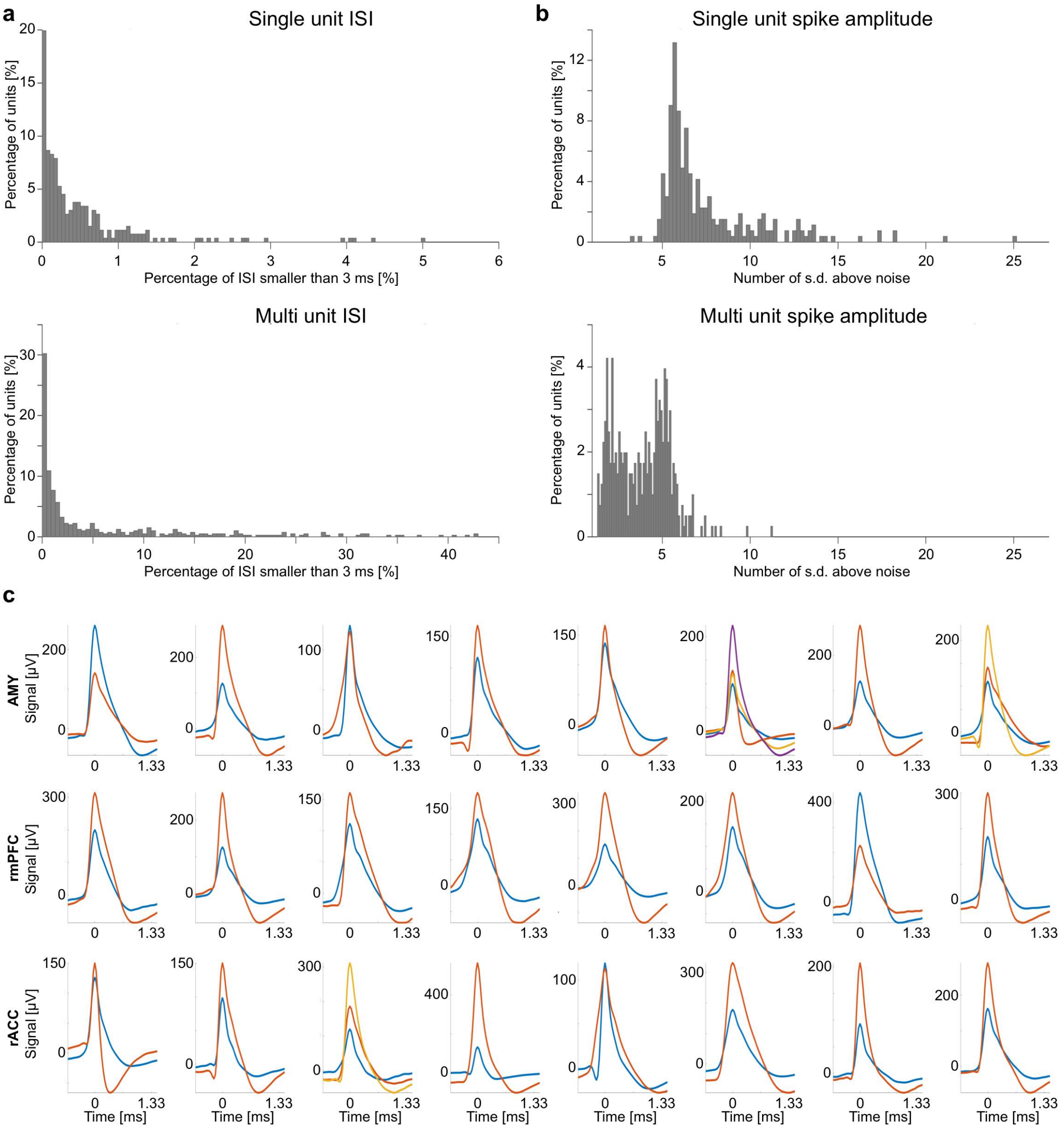

Supplementary Figure 3 | Single-unit characteristics. Population statistics for the differentiation of single-units and multi-units within the recorded data. (**a**) Histograms of the percent of all interspike intervals (ISI), which were shorter than 3 ms for single-units (top) and multi-units (bottom), the median of the two distributions were 0.25% (single-units) and 1.4% (multi-units). (**b**) Histograms of Spike amplitudes (the number of s.d. that the peak of the mean spike waveform was above the noise level across all recorded spikes within a unit) for single-units (top) and multi-units (bottom). (**c**) Examples of multiple units recorded from within the same electrodes (one plot per electrode) in the AMY (top), rmPFC (middle) and rACC (bottom). Separation between units was done manually by an expert using wave_clus[36], making sure that individual spike waveforms differed qualitatively strongly from each other.

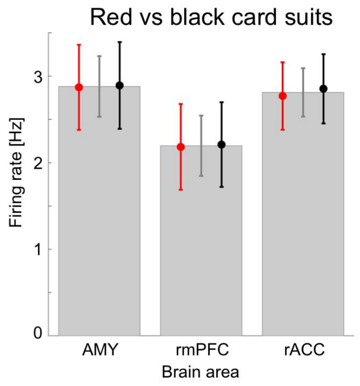

Supplementary Figure 4 | Absence of encoding of low-level card attributes. Histogram of the mean +/- s.e.m. firing rate during task performance (when the cards appeared on the table at the beginning of each round, grey). No significant difference in the firing rate was found, when comparing red card suits (hearts and diamonds) to black suits (clubs and spades; mean +/- s.e.m in red and black for each brain area respectively; p(AMY) = 0.771, p(rmPFC) = 0.71, p(rACC) = 0.157 > 0.05/3, t-tests).

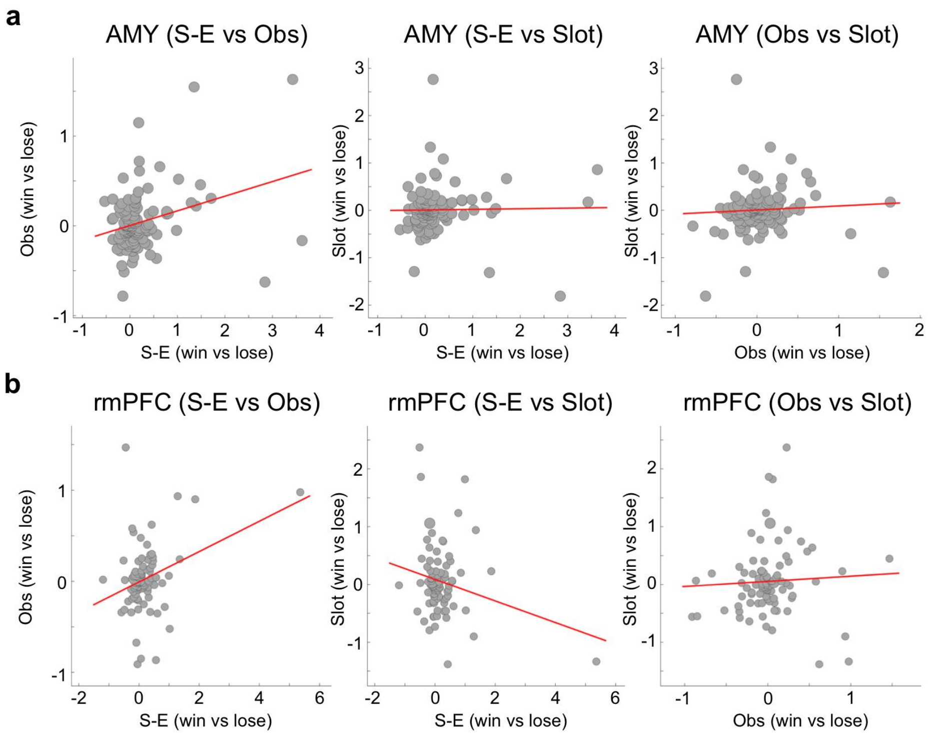

Supplementary Figure 5 | Response difference correlations in AMY and rmPFC. Pearson correlation analysis between trial types as in Fig. 3c but for the AMY and the rmPFC. Every data point represents the mean response difference between winning and losing for the respective trial type in a single neuron (in the rmPFC there were two identical values, which are here represented by a larger marker size than the remaining data points). (**a**) In the AMY the correlation analysis yielded p(S-E *vs* Obs) $< 10^{-4} < 0.01$, r(S-E *vs* Obs) = 0.307; p(S-E *vs* Slot) = 0.847 > 0.01, r(S-E *vs* Slot) = 0.017; and p(Obs *vs* Slot) = 0.502 > 0.01, r(Obs *vs* Slot) = 0.061. (**b**) In the rmPFC the results were p(S-E *vs* Obs) < 0.001 < 0.01, r(S-E *vs* Obs) = 0.33; p(S-E *vs* Slot) = 0.031 > 0.01, r(S-E *vs* Slot) = 0.222; and p(Obs *vs* Slot) = 0.116 > 0.01, r(Obs *vs* Slot) = 0.605.

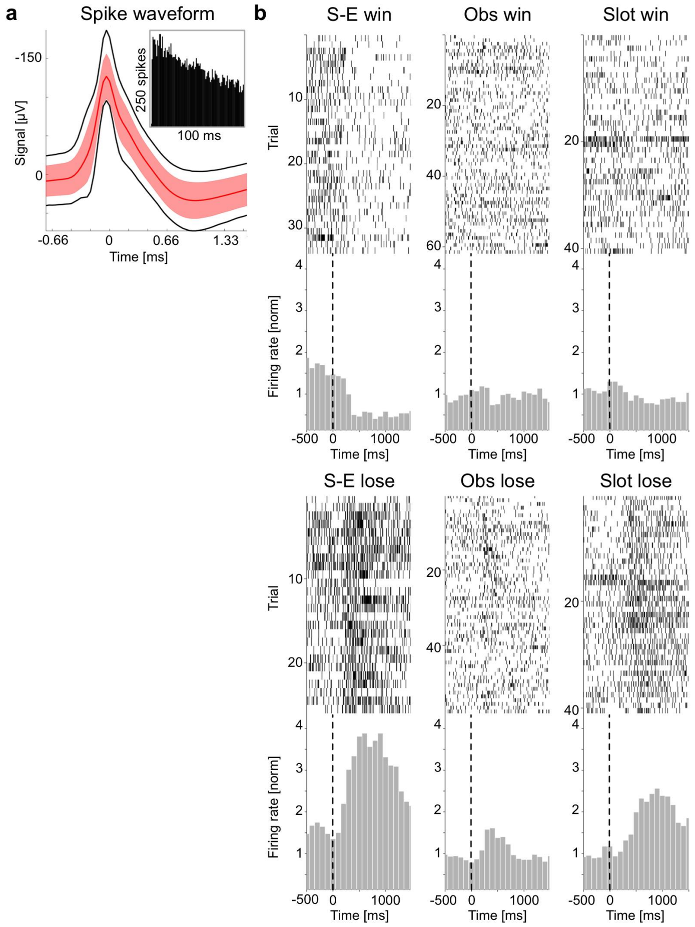

Supplementary Figure 6 | Example neuron. (**a**) Waveform of all spikes recorded from a single rACC neuron presented as in Fig. 3d (n = 26406). (**b**) For the same neuron as in (a) raster plots and peristimulus time histograms of wins (top) and losses (bottom) in self-experienced (left), observed (middle), and slot machine trials (right). This example neuron responded differentially to winning and losing in all three trial types, showing a higher firing rate for losses than for wins independent of whether the trial was self-experienced, observed or whether it was a slot machine trail.

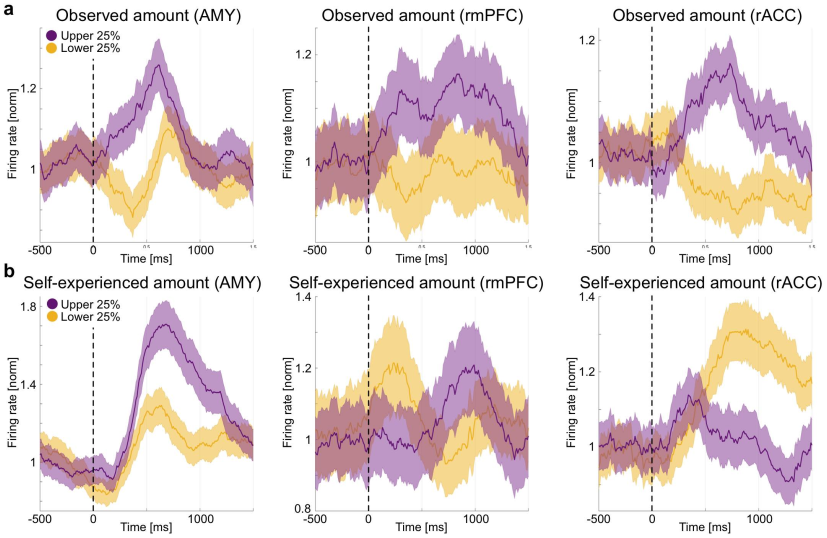

Supplementary Figure 7 | Firing rates during amount encoding. Normalized mean firing rates across all selected neurons (n_{AMY} = 30, n_{rmPFC} = 25, n_{rACC} = 40) for the upper and lower quartiles of trials ordered according to the amount won or lost during observed (a) and self-experienced trials (b). Note how in the AMY (and to a limited extent in the rmPFC) trials with higher amount values on average also display a higher firing rate for observed and self-experienced trials. In the rACC, however, this is only true for observed trials while in self-experienced trials the relationship is inverted, as trials with higher amount values on average display a lower firing rate.

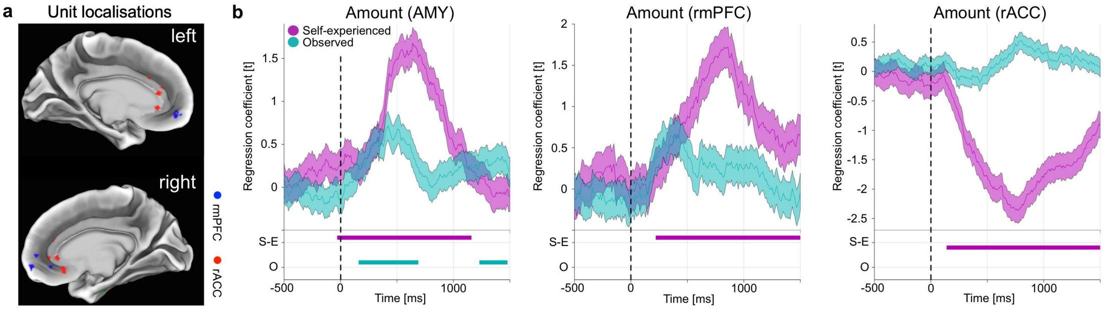

Supplementary Figure 8 | Encoding of amount when selecting for self-experienced trials. (**a**) Localization of the recording sites of the neurons selected in the analysis described in Fig. 4 (MNI space, see Supplementary Table 1). (**b**) The same analysis and presentation as in Fig. 4a but for neurons with a positive regression coefficient for self-experienced amounts in the AMY and the rmPFC, and a negative regression coefficient for self-experienced amounts in the rACC. In the AMY (left, n = 40) and the rmPFC (middle, n = 21) the selected neurons showed the same positive mean regression coefficient for observed outcomes as to self-experienced outcomes, although this effect was only found to be significant in the AMY (α = 0.01). In the rACC (right, n = 51) an opposite, positive mean coefficient to observed outcomes was measured, although, as in the rmPFC, this effect was not significant (α = 0.01).

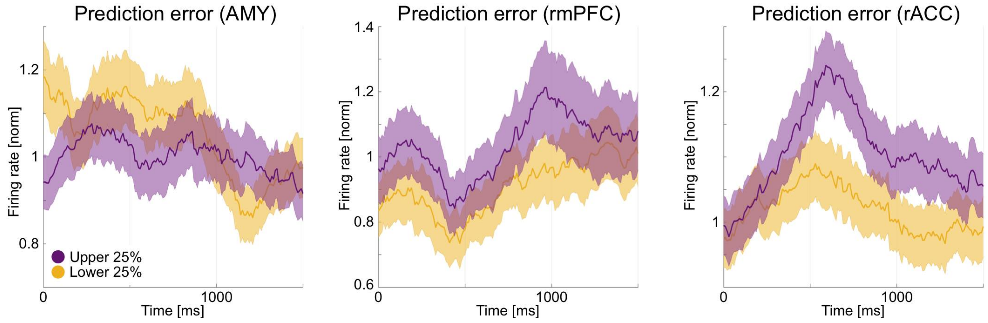

Supplementary Figure 9 | Firing rates during PE encoding. Normalized mean firing rates across all selected neurons ($n_{AMY} = 14$, $n_{rmPFC} = 9$, $n_{rACC} = 22$) for the upper and lower quartiles of trials ordered according to the PE during observed trials. Note how in the rACC trials with higher PE values on average also display a higher firing rate.

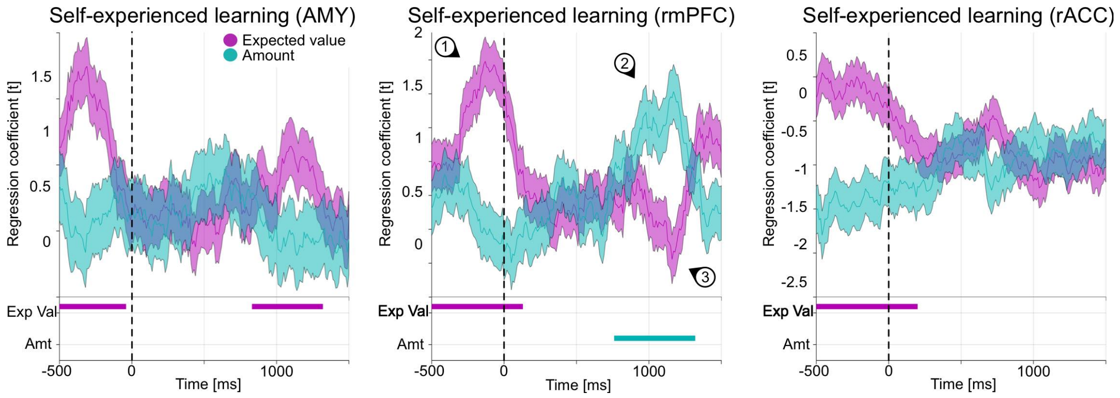

Supplementary Figure 10 | Encoding of learning parameters when selecting for self-experienced trials. The same analysis and presentation as in Fig. 5a but for neurons selected for encoding self-experienced expected value during choice in the AMY (left, n = 21), the rmPFC (middle, n = 7) and the rACC (right, n = 18). The neuronal populations in the three brain areas did not encode a self-experienced PE at outcome (the conjunction of a significant positive encoding of amount and a significant negative encoding of the expected value). In the rmPFC, however, in addition to the selected-for positive regression to the expected value at choice (①) and a significant positive regression to the amount (②,$\alpha = 0.01$), we did observe a simultaneous non-significant negative regression to the expected value during the outcome period (③,$\alpha = 0.01$), suggesting a possible trend for self-experienced PE encoding in the rmPFC. We note that the statistical power in this analysis was lower than in the observed learning analysis of Fig. 5a for the following three reasons: (1) the subjects only played half the number of trials compared to the two observed players (Obs_1 and Obs_2, see Fig. 1a); (2) For the choices made by the subjects, the observed trials were more recent than the self-experienced trials and may therefore have been weighted differently; (3) the number of neurons returned by our selection criterion was consistently lower in the rmPFC than in the rACC.

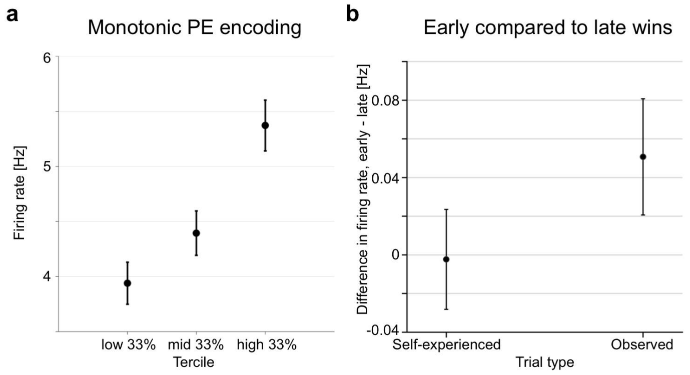

Supplementary Figure 11 | Model-free analysis of prediction errors in the rACC. (**a**) Mean (+/- s.e.m.) firing rate in lower, middle and upper tercile of trials sorted according to their PE values across all rACC neurons from Fig. 5. Note the positive monotonic relationship between the mean PE values and the firing rate. (**b**) The mean (+/- s.e.m) difference in firing rate between matched wins that occurred early and late within a game (early – late $10 wins; early – late $100 wins) for the subpopulation of single rACC neurons that showed a group observational PE response (Fig. 5). Differences are plotted separately for self-experienced and observed trial outcomes.

**Region**	**AMY**		**rmPFC**		**rACC**	
**Hemisphere**	L	R	L	R	L	R
**Coordinates (MNI 152 1mm^3 Template)**	**-19 -5 -16**	***20 -4 -17***	**-11 53 -8**	***8 54 3***	***-5 34 13***	**7 42 0**
	**-14 -6 -17**	**20 -7 -21**	**-5 51 -6**	***6 40 -8***	***-4 25 28***	**5 34 1**
	-16 -7 -21	*20 -1 -23*	*-3 50 5*	**5 59 -8**	**-3 33 -3**	***4 27 -12***
	-15 -3 -20	*17 5 -23*	-6 51 11	*5 53 -10*	-6 43 11	***1 38 20***
	-21 -4 -19	*17 0 -22*	-2 42 -6	7 37 -14	-4 32 31	*5 30 20*
	-17 -3 -18	23 5 -24	-2 43 -10	*2 49 -11*	-3 33 31	11 24 38
		20 -10 -15				7 36 13

Supplementary Table 1 | Localization of microelectrodes, Table lists the coordinates of localized microelectrodes analysed in the study in standardized Montreal Neurological Institute (MNI) space (resolution = 1mm^3). Bold coordinates denote localisation of units contributing to AMT encoding plots (Figure 4, S7). Italicised coordinates denote those units contributing to observational PE encoding plots (Figure 5). Those units included in both plots are shown in bold italics.
